# Postoperative Outcomes of Enucleation and Standard Resections in Patients with a Pancreatic Neuroendocrine Tumor

**DOI:** 10.1007/s00268-015-3341-9

**Published:** 2015-11-25

**Authors:** Anneke P. J. Jilesen, Casper H. J. van Eijck, Olivier R. C. Busch, Thomas M. van Gulik, Dirk J. Gouma, Els J. M. Nieveen van Dijkum

**Affiliations:** Department of Surgery, Academic Medical Center, Meibergdreef 9, PO Box 22660, 1105 AZ Amsterdam, The Netherlands; Department of Surgery, Erasmus Medical Center, Rotterdam, The Netherlands

## Abstract

**Background:**

Either enucleation or more extended resection is performed to treat patients with pancreatic neuroendocrine tumor (pNET). Aim was to analyze the postoperative complications for each operation separately. Furthermore, independent risk factors for complications and incidence of pancreatic insufficiency were analyzed.

**Methods:**

Retrospective all resected patients from two academic hospitals in The Netherlands between 1992 and 2013 were included. Postoperative complications were scored by both ISGPS and Clavien–Dindo criteria. Based on tumor location, operations were compared. Independent risk factors for overall complications were identified. During long-term follow-up, pancreatic insufficiency and recurrent disease were analyzed.

**Results:**

Tumor enucleation was performed in 60/205 patients (29 %), pancreatoduodenectomy in 65/205 (31 %), distal pancreatectomy in 72/205 (35 %) and central pancreatectomy in 8/205 (4 %) patients. Overall complications after tumor enucleation of the pancreatic head and pancreatoduodenectomy were comparable, 24/35 (69 %) versus 52/65 (80 %). The same was found after tumor enucleation and resection of the pancreatic tail (36 vs.58 %). Number of re-interventions and readmissions were comparable between all operations. After pancreatoduodenectomy, 33/65 patients had lymph node metastasis and in patients with tumor size ≤2 cm, 55 % had lymph node metastasis. Tumor in the head and BMI ≥25 kg/m^2^ were independent risk factors for complications after enucleation. During follow-up, incidence of exocrine and endocrine insufficiency was significant higher after pancreatoduodenectomy (resp. 55 and 19 %) compared to the tumor enucleation and distal pancreatectomy(resp. 5 and 7 % vs.8 and 13 %). After tumor enucleation 19 % developed recurrent disease.

**Conclusion:**

Since the complication rate, need for re-interventions and readmissions were comparable for all resections, tumor enucleation may be regarded as high risk. Appropriate operation should be based on tumor size, location, and functional status of the pNET.

## Introduction

Type of operation in patients with a pancreatic neuroendocrine tumor (pNET) primarily depends on tumor location and tumor size [1, 2]. Besides a standard pancreatic resection, such as pancreatoduodenectomy or distal pancreatectomy, enucleation of a tumor in pancreatic head or corpus/tail is frequently performed in patients with well-defined, small pNET located away from the pancreatic duct. Compared to pancreatic adenocarcinoma, pNETs have a much better prognosis after resection and therefore, long-term postoperative outcome and pancreatic function may be more relevant in these patients [2, 3].

Owing to the introduction of clear clinical grading systems for pancreatic fistula, postoperative bleeding and delayed gastric emptying by the International Study Group of Pancreatic Surgery (ISGPS) [4–6], the severity of these postoperative complications can now be categorized in detail. Also using the Clavien–Dindo grading system, the severity of the complications can be specified in terms of need for re-intervention and organ failure [7].

In the most studies on postoperative outcome, patients with different diagnoses are enrolled. This may affect the outcome since the diagnosis “pancreatic neuroendocrine tumor” is in itself a risk factor for developing pancreatic complications, especially pancreatic fistula [8]. pNET are often associated with a non-dilated pancreatic duct and subsequently less inflammation and stromal changes in the pancreatic parenchyma, which leads to a soft and friable pancreas during surgery. These factors consequently increase the pancreatic fistula rate after resection [8–14]. Furthermore, tumor enucleation is often compared with a “standard resection” while a standard resection carries no uniform definition. Besides patients with a pancreatoduodenectomy or distal pancreatectomy, studies included patients with total pancreatectomy or even central pancreatectomy [15–17].

Aim of this study was to analyze postoperative complications for different operations, in particular enucleation versus standard resections, using both the ISGPS and Clavien–Dindo grading system in patients with a pancreatic neuroendocrine tumor. Furthermore, we identified risk factors for overall complications after resection. Finally, the rate of pancreatic insufficiency was analyzed for the operations assessed in this study.

## Methods

### Patients

All patients with resected pNET were included from the Academic Medical Center (AMC) in Amsterdam and from the Erasmus Medical Center (Erasmus MC) in Rotterdam, both in The Netherlands, in the period January 1992 through December 2013. Both tertiary centers are high-volume centers for pancreatic surgery and are also specialized in the treatment of neuroendocrine tumors. Patients with extended combined operations were excluded. Eligible patients were identified from pathology reports of all pancreatic resections. Patient and operation characteristics, postoperative complications, and mortality were extracted from the patient records. Based on the operation, patients were stratified in different groups. A hereditary syndrome is defined as multiple endocrine neoplasia (MEN) syndrome or Von Hippel–Lindau syndrome.

### Pancreatic operations

#### Pancreatic enucleation

For small, superficial neuroendocrine tumors without a connection or in close relation to the pancreatic duct a pancreatic enucleation was performed while attempting not to damage the pancreatic duct [18]. The decision was made after imaging and during surgery based on tumor size, superficial location, and the relation to the pancreatic duct. In none of the patients with an enucleation, lymphadenectomy was carried out. Laparoscopic tumor enucleation was introduced in the most recent years of the study.

#### Pancreatic resection—pancreatoduodenectomy

For tumors located in the head of the pancreas, a pylorus-preserving pancreatoduodenectomy (PPPD) or, if necessary, a classical Whipple-Kausch pancreatoduodenectomy was performed as described previously [19, 20]. Reconstruction was carried out through an end-to-side pancreaticojejunostomy (PJ), end-to-side hepaticojejunostomy (HJ), and a gastrojejunostomy (GJ) or duodenojejunostomy (DJ) [21]. One silicon drain was left in the lesser sac [19].

#### Pancreatic resection—distal pancreatectomy

For tumors located in the body or the tail of the pancreas, a distal pancreatectomy, with or without a splenectomy, was the standard. Splenectomy was performed to ensure a radical resection in case of a suspected malignant tumor or an inadequate remaining blood supply to the spleen [22]. In all patients, the pancreatic remnant was closed either with sutures or a stapler. In the most recent years, laparoscopic tail resection was introduced.

#### Pancreatic resection—central pancreatectomy

A central pancreatectomy was performed for tumors located in the central part of the pancreas not suitable for enucleation. Central pancreatectomy was performed when the tumor was embedded deeply in the pancreatic tissue with risk of damage to the pancreatic duct. Reconstruction was performed using an end-to-side, duct-to-mucosa PJ to secure internal drainage of the pancreatic tail remnant. The proximal pancreatic stump was closed with sutures or a surgical stapler. Central pancreatectomy enables the surgeon to perform a less extended, parenchyma preserving resection compared to a complete corpus/tail resection [23, 24].

### Peptide receptor radionuclide therapy (PRRT)

A specific group of patients (*n* = 12) from Erasmus MC, with locally advanced pNET or hematogenous metastasis, received preoperative treatment with PRRT before the index operation [25]. After PRRT, patients had partial/complete response of their primary tumor or metastases and therefore were considered for resection.

### Perioperative treatment with somatostatin analogs

Somatostatin analogs were routinely administered to patients with a high risk of developing pancreatic fistula, i.e., patients with a non-dilated pancreatic duct and/or soft pancreatic tissue except in patients with insulinomas [13, 26]. Also patients with symptoms related to their NET may have been treated with a somatostatin analog. The diameter of the main pancreatic duct (MPD) was scored as <3 or ≥3 mm based on radiological description on CT images or on perioperative findings, using a cut-off value of ≥3 mm for a dilated duct [27, 28].

### Postoperative complications

The primary endpoint was the overall complication rate after pancreatic surgery.

Complications were identified from the patient records. Also the discharge letters and medical notes were checked for reported complications. The medication list was checked on the use of antibiotics and somatostatin analogs. Laboratory tests and additional endoscopic or radiological imaging or interventions during hospitalization were reviewed to confirm whether they were performed to detect or treat complications. The independent Dutch national surgical complication registry used in both surgical departments was consulted for possibly overlooked complications [29]. All pathology specimens were reviewed and if necessary rescored according the most recent WHO classification of 2010 [30].

Severity of major complications after pancreatic surgery was scored using both the ISGPS and the Clavien–Dindo classification [7]. These major complications included: pancreatic fistula grade B/C, delayed gastric emptying grade B/C and postoperative bleeding grade B/C [4–6]. Grade A complications were not considered as major complications since they had no clinical consequences. In addition, other complications were recorded. Chylous ascites was defined as a drain output with milky appearance, occurring simultaneously with the start of enteral feeding and confirmed with elevated triglyceride in the drain output as reported previously [31]. Pneumonia, surgical site infection, intra-abdominal abscess, and urinary tract infection were defined according to the Centers of Disease Control and Prevention (CDC) guidelines [32–34]. In this study, pneumonia was described as hospital-acquired pneumonia. Readmission was understood to be a new admission within 30 days after discharge from the initial hospitalization. Re-intervention was understood to be a surgical, endoscopic, or radiological re-intervention. Tumors were divided into small (≤2 cm) and large (>2 cm) tumors, based on preoperative radiological imaging. Resection margins are classified according the Royal College of Pathologists [35] whereby tumors with microscopic margin involvement <1 mm are classified as R1. Also in patients with unclear margins due to the damage caused by coagulation are classified as R1. Lymph node metastases in the resected specimen were proven by pathology.

### Long-term follow-up

Secondary endpoint was pancreatic insufficiency during follow-up. Exocrine insufficiency was defined as the persisting use of pancreatic enzymes as treatment for steatorrhea at least 6 months after surgery. Endocrine insufficiency was defined as the development of diabetes mellitus after surgery. Patients with preexisting diabetes mellitus or preexisting exocrine insufficiency were excluded from the analysis.

### Data analyses

Statistical analysis was performed using IBM SPSS statistics version 20.0 (IBM Corp. Armonk, NY: IBM Corp.). Based on the type of outcome data, the unpaired *t* test, Mann–Whitney *U* test or the *χ*^2^ d test, was used. The continuous variables are described with the interquartile range (IQR) or range. Postoperative outcome was compared in two different groups; patients with a tumor enucleation of the pancreatic head were compared with patients with pancreatoduodenectomy, and patients with a tumor enucleation of pancreatic corpus/tail were compared with a distal pancreatectomy. A univariable analysis was performed to identify risk factors for overall complications. The factors with a *p* value <0.1 were analyzed in the multivariable logistic regression analysis to identify independent risk factors. A *p* value below 0.05 was considered significant. Missing values were imputed using multiple imputations [36]. The group of patients with a central pancreatectomy (*n* = 8) was too small to perform statistical testing, therefore only the exact numbers are displayed in tables. Long-term follow-up was presented as median in months.

## Results

Overall 10 patients were excluded since they underwent extended combined operations. One patient underwent a liver transplantation and distal pancreatectomy simultaneously, two patients underwent hemihepatectomy and distal pancreatectomy simultaneously and four patients received both wedge resection of liver metastasis and radiofrequency ablation (RFA) of liver metastasis in combination with a distal pancreatectomy. The other three patients were excluded because resection was atypical; total pancreatectomy in two patients and duodenum preserving pancreatic head resection in one patient. Finally, a total of 205 patients with pNET with mean age of 52.5 years were included, 93 patients (45 %) were male and 112 were female (55 %). Sixty were treated with tumor enucleation, 65 with pancreatoduodenectomy, 72 with distal pancreatectomy, and 8 with a central pancreatectomy. Characteristics of the different groups of patients are listed in Table 1.

**Table 1:** Characteristics of patients with a resected pancreatic neuroendocrine tumor

	Tumor enucleation Pancreatic head	Pancreatoduodenectomy (PD)	Enucleation head versus PD	Tumor enucleation of pancreatic corpus/tail	Distal pancreatectomy (DP)	Enucleation corpus/tail versus DP	Central pancreatectomy
	*n* = 35	*n* = 65	*p* value	*n* = 25	*n* = 72	*p* value	*N* = 8
Age, mean (SD)	50 (15.5)	55 (10.7)	0.07	50 (13.4)	51 (15.6)	0.70	57 (10.3)
Male, *n* (%)	17 (49)	37 (57)	0.42	9 (36)	26 (36)	1.00	4 (50)
BMI, mean (SD)	26.0 (4.3)	25.4 (3.9)	0.49	25.6 (3.7)	25.1 (2.9)	0.44	25.8 (2.4)
ASA classification *n* (%)							
I	12 (34)	18 (28)	0.58	9 (36)	21 (29)	0.70	2 (25)
II	19 (54)	42 (65)		14 (56)	47 (65)		5 (63)
III	4 (12)	5 (7)		2 (8)	4 (6)		1 (12)
Perioperative treatment with Somatostatin analog, *n* (%)	11 (31)	30 (46)	0.15	10 (40)	14 (19)	<0.05*	2 (25)
Hereditary syndrome, *n* (%)^a^	2 (6)	2 (3)	0.52	–	10 (14)	<0.05*	–
Dilated main pancreatic duct, *n* (%)	2 (6)	26 (40)	<0.01*	1 (4)	9 (13)	0.23	1 (12)
Preoperative PRRT, *n* (%)	–	9 (14)	<0.05*	–	3 (4)	0.30	--
Diabetes Mellitus, *n* (%)	1 (3)	6 (9)	0.23	–	8 (11)	0.08	0
Nonfunctional tumor, *n* (%)	9 (26)	60 (92)	<0.01*	7 (28)	46 (64)	<0.01*	8 (100)
Diameter tumor size in mm, median (IQR)	13 (10–15)	40 (25–50)	<0.01*	16 (11–22)	25 (13–50)	<0.05*	22 (14–36)

*SD* standard deviation, *BMI* body mass index, *ASA* American Society of Anesthesiologists, *PRRT* peptide receptor radionuclide therapy, *IQR* interquartile range

* *p* value <0.05

^a^Multiple endocrine neoplasia syndrome or Von Hippel–Lindau syndrome

### Perioperative outcomes

The mean operation time for tumor enucleation was 200 min, significantly shorter than that for pancreatoduodenectomy or distal pancreatectomy, i.e., 403 and 260 min. In total, 35/60 (58 %) pNET treated with tumor enucleation were located in the pancreatic head of which 2/35 were operated laparoscopically with one conversion. The other 25 patients had a tumor enucleation of the pancreatic corpus/tail, nine patients were operated laparoscopically with four conversions to an open procedure. Seventeen of the 72 distal pancreatectomies were laparoscopic operations, and 8 of these (47 %) were converted to an open operation.

### Postoperative outcomes

Overall, 132/205 patients (64 %) had one or more complications. The complication rates after tumor enucleation of the head versus pancreatoduodenectomy were not significant different with 69 and 80 %, respectively (*p* = 0.20) (Table 2). The overall complication rates after tumor enucleation of corpus/tail versus distal pancreatectomy were 36 and 58 %, respectively (*p* = 0.054).

**Table 2:** Postoperative outcome after pancreatic resection

	Enucleation head	Pancreatoduodenectomy (PD)	Enucleation	Tumor enucleation of pancreatic corpus/tail	Distal pancreatectomy (DP)	Enucleation corpus/tail versus DP	Central pancreatectomy
	*n* = 35	*n* = 65	*p* value	*n* = 25	*n* = 72	*p* value	*N* = 8
Overall complications, n (%)	24 (69)	52 (80)	0.20	9 (36)	42 (58)	0.054	5 (62)
In-hospital stay in days, median (range)	12 (5–136)	15 (7–88)	0.10	9 (2–147)	11 (3–74)	< 0.01*	11 (9–97)
Grade B/C complications after pancreatic surgery, *n* (%)	16 (46)	25 (39)	0.48	6 (24)	11 (15)	0.32	4 (50)
Pancreatic fistula	14 (40)	9 (14)	<0.01*	5 (20)	7 (10)	0.18	3 (38)
Postoperative bleeding	1 (3)	7 (11)	0.16	1 (4)	5 (7)	0.60	–
Delayed gastric emptying	9 (26)	14 (22)	0.64	–	–	NIA	2 (25)
Clavien–Dindo grading of grade B/C complications, *n* (%)							
Grade I	–	–	NIA	–	–	NIA	–
Grade II	4 (25)	3 (12)	0.28	1 (17)	1 (9)	0.64	–
Grade IIIa	8 (50)	13 (52)	0.90	3 (50)	4 (36)	0.59	3 (60)
Grade IIIb	3 (19)	3 (12)	0.55	1 (17)	6 (55)	0.13	1 (20)
Grade IVa	0 (–)	2 (8)	0.25	0 (–)	0 (–)	NIA	–
Grade IVb	1 (6)	2 (8)	0.83	1 (17)	0 (–)	0.16	–
Grade V	0	2 (8)	0.25	–	–	NIA	–
Readmission within 30 days, n (%)	5 (14)	15 (23)	0.30	2 (8)	7 (10)	0.80	3 (38)
Overall need for re-interventions, n (%)	17 (49)	30 (46)	0.82	6 (24)	14 (19)	0.63	4 (50)
Endoscopic	10 (29)	15 (23)	0.55	2 (8)	0	**<**0.05*	2 (25)
Radiological	9 (26)	15 (23)	0.77	2 (8)	7 (10)	0.80	2 (25)
Surgical	1 (3)	8 (12)	0.12	2 (8)	7 (10)	0.80	1 (13)
Other complications, *n* (%)	6 (17)	22 (34)	0.08	0	10 (14)	<0.05*	–
Clavien–Dindo grade III-V of other complications, *n* (%)	1 (17)	6 (27)	0.60	–	3 (11)	0.30	–
In-hospital mortality	–	3 (5)	0.20	–	–	NIA	–

*NIA* not in analysis

* *p* value <0.05

**Fig. 1:**
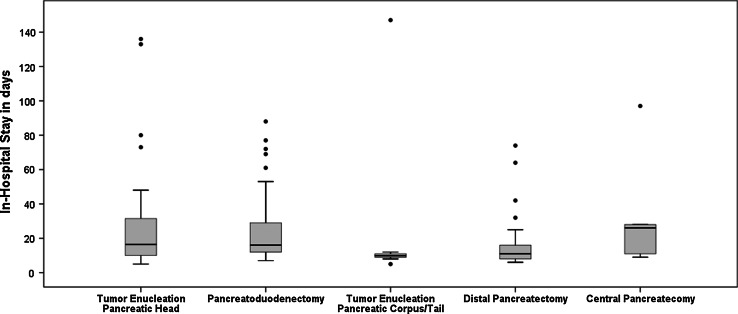
In-hospital stay in patients with complications for each operation separately. • outlier patients

Median in-hospital stay was 12 days (IQR 9–17). The median in-hospital stay was significantly prolonged in patients with complications (14 days IQR 10–25) compared to patients without complications (10 days, IQR 7–13), *p* < 0.001. In patients with complications, the in-hospital stay was not significantly different after tumor enucleation of pancreatic head, i.e., 17 days (range 5–136) compared to 16 days after pancreatoduodenectomy (range 7–88 days) *p* = 0.73. As depicted in Fig. 1, the interquartile range of the in-hospital stay after tumor enucleation of the pancreatic head and pancreatoduodenectomy was broad: i.e., 10–33 days and 12–30 days. Most patients with complications were discharged from the hospital within 21 days, but a considerable number of patients had a prolonged length of hospital stay. Nine of the 35 (26 %) patients after tumor enucleation of the pancreatic head, and 18/65 (28 %) patients after pancreatoduodenectomy were admitted to the hospital ≥21 days (*p* = 0.8). In addition, there were some extreme outliers consisting patients with an in-hospital stay of > 80 days due to complications. In-hospital stay in 9/25 (36 %) patients with complications after tumor enucleation of the pancreatic corpus/tail and 42/72 (58 %) patients after distal pancreatectomy were comparable, i.e., 10 days (range 5–147) versus 11 days (range 3–74) *p* = 0.41.

Significantly more patients suffered from clinically relevant pancreatic fistula (grade B/C) after tumor enucleation of pancreatic head (40 %) compared to pancreatoduodenectomy (14 %), *p* < 0.01. The severity of the major complications based on the Clavien-Dindo classification was not significantly different between the different operations (see Table 2). Overall, 62/205 patients (30 %) had a grade B/C complication according to the ISGPS criteria, e.g., pancreatic fistula, postoperative bleeding or delayed gastric emptying. Based on the Clavien-Dindo grading system severity cut-off value of ≥3, 15 % of the patients (9/62) with a grade B/C complication were missed. In these 9 patients with a grade B/C pancreatic fistula, the perioperative drain was used for drainage of abscesses, and therefore there was no need for a re-intervention. These nine patients had a Clavien–Dindo severity score grade II. Although the number of operated patients increased over time, the incidence of major complications remained the same.

The number of readmissions within 30 days after discharge was 14 % (*n* = 5) after tumor enucleation of pancreatic head compared to 23 % (*n* = 15) after pancreatoduodenectomy (*p* = 0.30). The number of readmissions after tumor enucleation of corpus/tail was 8 % (*n* = 2) compared to 10 % (*n* = 10) in patients after distal pancreatectomy (*p* = 0.80).

Rates of re-interventions and readmissions did not differ between tumor enucleation of the pancreatic head (49 %) and pancreatoduodenectomy (46 %) *p* = 0.82 or between enucleation of pancreatic corpus/tail tumors (24 %) and distal pancreatectomy (19 %) *p* = 0.63. Also after further subdivision in endoscopic, radiological, and surgical re-interventions, no significant differences were found between the operations.

The incidence of other, miscellaneous complications was higher after pancreatoduodenectomy (34 %) compared to tumor enucleation of pancreatic head (17 %) *p* = 0.08 and significantly higher after distal pancreatectomy (14 %) compared to tumor enucleation of pancreatic corpus/tail (0 %) *p* < 0.05. The most frequent other complications were chylous ascites in 9 patients, pneumonia in 6 patients, and urinary tract infection in 6 patients.

In-hospital mortality was 1.5 % (3/205); all deaths were related to pancreatoduodenectomy. One patient died after an unexpected and unsuccessful attempt at cardiopulmonary resuscitation after a cardiac arrest. The patient had no signs of abdominal sepsis or multiple organ failure. The second patient died due to respiratory insufficiency after an aspiration pneumonia associated with abdominal sepsis and pancreatic fistula. The third patient died due to cardiac complications after extensive perioperative blood loss in combination with a medical history of tricuspid insufficiency and mitral valve replacement.

### Risk factors for overall complications

A univariable analysis was performed to identify risk factors for overall complications of the three different types of resections, see Table 3. Regarding enucleation, a tumor located in the pancreatic head and BMI >25 kg/m^2^ were significant risk factors in the univariable analysis and in the multivariable analysis both factors were independent risk factors with an odds ratio of, respectively, 4.4 (95 %CI 1.4–11.2) and 3.5 (95 %CI 1.1–11.0). Regarding pancreatoduodenectomy, no risk factors were found in the univariable analysis, and therefore no multivariable analysis was performed. Regarding distal pancreatectomy, BMI >25 kg/m^2^ and diabetes mellitus were risk factors in the univariable analysis but in the multivariable analysis no independent risk factors were found.

**Table 3:** Univariable analysis to identify risk factors for overall complications after resection for pancreatic neuroendocrine tumor

	Enucleation			Pancreatoduodenectomy			Distal pancreatectomy		
	Complications (*N* = 33)	No complications (*N* = 27)	*p* value	Complications (*N* = 52)	No complications (*N* = 13)	*p* value	Complications (*N* = 42)	No complications (*N* = 30)	*p* value
Age, mean (SD)	52.8 (12.6)	46.6 (16.1)	0.102	55.69 (10.8)	54.62 (10.8)	0.749	50.7 (15.04)	52.3 (16.69)	0.677
Male, *n* (%)	17 (52)	9 (33)	0.157	31 (60)	6 (46)	0.381	14 (33)	12(40)	0.561
BMI ≥25 kg/m^2^	21 (64)	10 (37)	0.04*	24 (46)	6 (46)	1.00	26(50)	12(40)	0.066
ASA classification >II, *n* (%)	5 (15)	1 (4)	0.141	5 (10)	0	0.245	1 (2)	3(10)	0.164
Diabetes mellitus, *n* (%)	0	1 (4)		5 (10)	1 (8)	0.830	7 (17)	1 (3)	0.076
Smoking, *n* (%)	13 (39)	9 (33)	0.628	16 (31)	2 (15)	0.268	5 (12)	2 (7)	0.460
Hereditary syndrome^a^, *n* (%)	1 (3)	1 (4)	0.885	1 (2)	1 (8)	0.281	7(17)	3 (10)	0.420
Tumor size >2 cm, *n* (%)	4 (12)	7 (26)	0.169	42 (81)	12 (92)	0.321	25 (60)	16 (53)	0.601
Preoperative treatment with Somatostatin analog	4 (12)	5 (19)	0.490	17 (33)	3 (23)	0.502	7 (17)	3 (10)	0.420
Dilated main pancreatic duct, *n* (%)	1 (3)	2 (7)	0.439	21 (40)	5 (38)	0.899	4 (10)	5 (17)	0.366
Preoperative PRRT, *n* (%)	–	–	–	7(13)	2(15)	0.857	2 (5)	1 (3)	0.765
Nonfunctional pNET, *n* (%)	9 (27)	6 (22)	0.653	48 (92)	12 (92)	1.00	30 (71)	16 (53)	0.115
Tumor in pancreatic head, *n* (%)	24 (73)	11 (41)	0.012*	–	–	–	–	–	–

*BMI* body mass index, *ASA* American Society of Anesthesiologists, *PRRT* peptide receptor radionuclide therapy, *pNET* pancreatic neuroendocrine tumor

* *p* value <0.05

^a^Patients with MEN1 or Von Hipple–Lindau syndrome

### Oncological outcome

Lymphadenectomy was not a standard part of the operation in patients with tumor enucleation. In patients with a lymphadenectomy, 46 patients had a tumor size ≤2 cm and 99 patients had a tumor size >2 cm. Patients with a larger tumor size had significantly more often lymph node metastasis: 38/99 patients with a tumor size >2 cm had lymph nodes metastasis (38 %) compared to 7/46 patients with a tumor size ≤2 cm (15 %), *p* = 0.005. This also applies for the 11/72 patients after distal pancreatectomy with lymph node metastasis. Patients with a tumor size >2 cm (*n* = 10/41) had significantly more often lymph node metastasis compared to patients with a tumor size ≤2 cm (*n* = 1/31), *p* = 0.013. After pancreatoduodenectomy, 33/65 patients had lymph node metastasis. 50 % of the patients with a tumor size >2 cm (*n* = 27/54) had lymph node metastasis compared to 55 % of the patients with a tumor size ≤2 cm (*n* = 6/11), *p* = 0.78. Excluding patients after tumor enucleation, patients with a tumor located in the pancreatic head had significantly more often lymph node metastasis (*n* = 33/65) compared to patients with a pNET in corpus/tail (*n* = 13/80), *p* < 0.001.

**Fig. 2:**
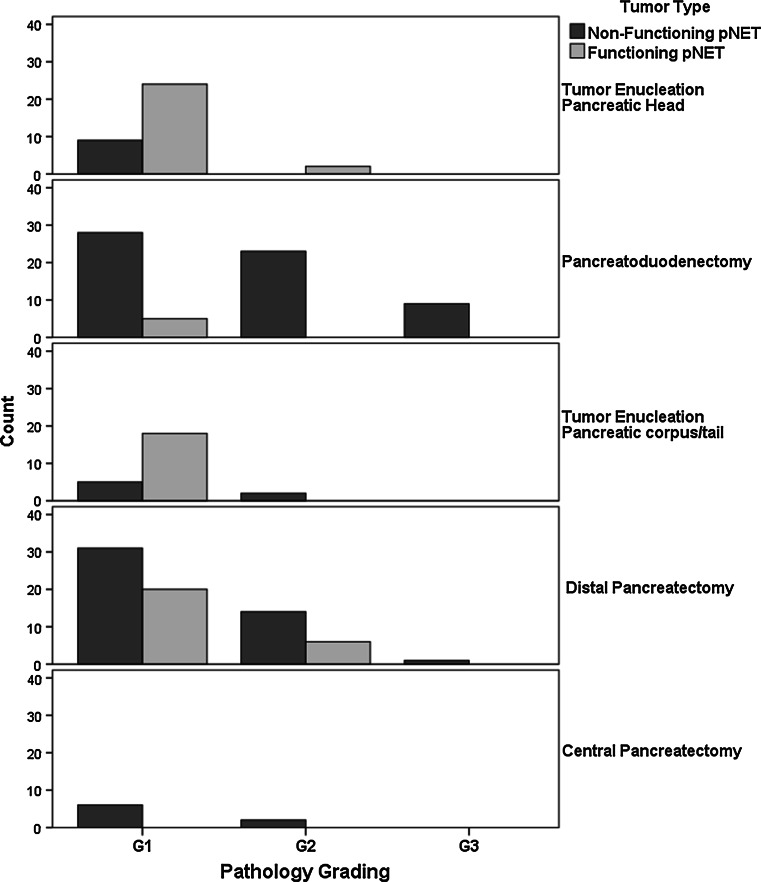
Distribution between pathology, operation, and tumor type after surgical resection in patients with pancreatic neuroendocrine tumor

Distribution of tumor grade, operations, and tumor functionality is displayed in Fig. 2. Compared with patients after pancreatoduodenectomy, significantly more patients had a tumor grade 1 in tumors enucleated from the pancreatic head, i.e., 51 versus 94 %, *p* < 0.01. Two patients (6 %) after tumor enucleation of the pancreatic head had a tumor grade 2. One patient had a tumor size of 10 mm and the other had a tumor size of 15 mm. After tumor enucleation in pancreatic corpus/tail, 23/25 of the patients had tumor grade 1 (92 %) compared to 51/72 patients after distal pancreatectomy (71 %), *p* < 0.05. Two patients (8 %) had a grade 2 pNET after tumor enucleation of pancreatic corpus/tail, both nonfunctional pNET (NF-pNET).

As depicted in Table 1, a tumor enucleation was often performed in patients with a functional pNET. Of the 26 patients with a functional pNET in the pancreatic head, 20 patients had an insulinoma (77 %) and six patients had a gastrinoma (23 %). Of the 18 functional pNET in the pancreatic corpus/tail, 17 (94 %) had an insulinoma and 1 patient had a gastrinoma. Only five patients had a functional pNET after pancreatoduodenectomy: 3 patients had a gastrinomas, 1 patients a VIPoma, and 1 patient a glucagonoma. In patients after distal pancreatectomy, 26/72 patients had a functional pNET. Of these 26 patients, 21 patients had an insulinoma, 3 patients a gastrinoma, 1 patient a VIPoma and 1 patient a glucagonoma. Of all insulinomas patients, only one patient had one positive lymph node in the resected specimen. This patient had a distal pancreatectomy with a tumor diameter of 2.5 cm.

Of the patients with a tumor size ≤2 cm, 86/95 (91 %) had a grade 1 tumor compared to 60/110 (55 %) patients with a tumor size >2 cm, *p* < 0.01. None of the pNETs with a tumor size <2 cm had a grade 3 tumor. All 10 grade 3 tumors were NF-pNET and the median tumor size was 43 mm (IQR 35–76). In patients with a tumor ≤1 cm (*n* = 14), all patients had a grade 1 tumor and 2 patients had lymph nodes metastasis (14 %). These two patients were diagnosed with gastrinoma.

R1 resection margins were more often found after tumor enucleation of pancreatic head (37 %) compared to pancreatoduodenectomy (17 %) *p* = 0.052. After tumor enucleation of corpus/tail and distal pancreatectomy, the differences were more comparable, i.e., 24 % versus 17 %, *p* = 0.50.

### Long-term follow-up

Functional follow-up was available for all included patients, with a median follow-up time of 29 months (IQR 10–64). Significantly more patients developed endocrine (19 %) and exocrine insufficiency (55 %) after pancreatoduodenectomy compared to tumor enucleation (7 and 5 %) or distal pancreatectomy (13 and 8 %) (*p* < 0.001), see Table 4. There were no significant differences between tumor enucleation and distal pancreatectomy regarding the rate of pancreatic insufficiency.

**Table 4:** Long-term outcome in patients with pancreatic neuroendocrine tumor after surgical resection

	Tumor enucleation (TE)	Pancreatoduodenectomy (PD)	Distal pancreatectomy (DP)	TE versus PD (*p* value)	TE versus DP (*p* value)	PD versus DP (*p* value)	Central pancreatectomy
All resected patients	*N* = 60	*N* = 65	*N* = 72				*N* = 8
Functional follow-up							
Exocrine insufficiency	3 (5)^a^	34 (55)^c^	6 (8)	<0.001	0.5	<0.001	0
Endocrine insufficiency	4 (7)^b^	11 (19)^d^	8 (13)^e^	0.05	0.3	0.3	2 (25)

^a^1 patient was excluded since preexisting exocrine insufficiency *n* = 59 patients were available for analysis

^b^1 patients was excluded since preexisting diabetes mellitus *n* = 59 patients were available for analysis; one of the 4 patients with endocrine insufficiency have had multiple pancreatic resections

^c^3 patients were excluded since postoperative mortality *n* = 62 patients were available for analysis

^d^7 patients were excluded since preexisting diabetes mellitus/postoperative mortality *n* = 58 patients were available for analysis

^e^8 patients were excluded since preexisting diabetes mellitus *n* = 64 patients were available for analysis; one of the 8 patients with endocrine insufficiency have had multiple pancreatic resections

Long-term oncologic follow-up was only available in patients with NF-pNET. However, 3/58 patients with an insulinomas developed tumor recurrence, all after distal pancreatectomy. Of the 130 patients with NF-pNET, 14 patients were excluded from long-term follow-up since 11 patients already had metastasis preoperatively and 3 patients died due to postoperative complications. Therefore, 116 patients with NF-pNET were available for long-term analysis. Median follow-up for recurrent disease was 37 months (IQR 18-60 months). Of the 116 patients, 16 patients have had a tumor enucleation, 50 patients a pancreatoduodenectomy, 42 patients a distal pancreatectomy, and 8 patients a central pancreatectomy. During oncological follow-up, 37 patients developed recurrent disease of which 15 patients died due to tumor progression. Of the 37 patients with recurrent disease, 68 % was proven by pathology (*n* = 25/37), the other 12 patients were proven by long-term follow-up. After enucleation, 19 % of patients developed recurrent disease (*n* = 3/16) of which 1 patient died. Tumor recurrence was significantly more frequent after pancreatoduodenectomy (*n* = 23) compared to tumor enucleation (*n* = 3) or distal pancreatectomy (*n* = 10). There were no significant differences in tumor recurrence or tumor related death between tumor enucleation and distal pancreatectomy.

## Discussion

This is the first study in patients with pancreatic neuroendocrine tumors which describes the postoperative outcomes of different operations separately using both ISPGS criteria and the Clavien–Dindo grading system. Besides the higher rate of grade B/C pancreatic fistula after tumor enucleation of pancreatic head, the postoperative outcomes were comparable between all operations. A tumor in the pancreatic head and a high BMI were independent risk factors for complications in patients after tumor enucleation. During follow-up, the rate of pancreatic insufficiency was significantly higher after pancreatoduodenectomy but there were no differences in the rate of pancreatic insufficiency after tumor enucleation or distal pancreatectomy.

The main complications after pancreatic surgery in patients with a pNET were pancreatic fistula grade B/C. The rate of these fistulas was the highest after tumor enucleation (31 %), especially after tumor enucleation in the pancreatic head (40 %). Other studies also report a high rate of pancreatic fistula after tumor enucleation [8, 37, 38]. In addition, due to the severe complications (pancreatic fistula, postoperative bleeding and delayed gastric emptying) the in-hospital stay, the need for re-interventions and readmissions were also comparable between tumor enucleation, pancreatoduodenectomy, and distal pancreatectomy. This is remarkable since tumor enucleation is often described as a minimally invasive operation with only an increased risk for pancreatic fistula. During follow-up, no differences were seen in the rate of pancreatic insufficiency between tumor enucleation and distal pancreatectomy. A high rate of pancreatic insufficiency was seen after pancreatoduodenectomy. Altogether, tumor enucleation can be regarded a high risk operation with considerable risk for postoperative morbidity. In fact, laparoscopic tail resection may be an attractive option compared to tumor enucleation [39, 40]. The learning curve of laparoscopic tail resection may be achieved faster than laparoscopic tumor enucleation. Laparoscopic tumor enucleation will probably be carried out less frequently since laparoscopic tumor enucleation is usually not indicated for malignant diseases. Further studies are needed to support this hypothesis.

The Clavien–Dindo grading system and the ISGPS criteria are both often used as scoring methods to describe the severity of postoperative complications [4–7]. In the Clavien–Dindo grading system, a cut-off value ≥ 3 is often used to describe severe complications [21, 27, 41, 42]. According to the ISGPS criteria, grade B/C complications are also severe complications. In our study, 15 % of the severe grade B/C complications were missed if only the Clavien–Dindo grading system with a cut-off ≤3 was used, especially in the analysis of pancreatic fistula. In some patients with a grade B/C pancreatic fistula, no additional re-intervention was needed, since the perioperative drain was used for abscess drainage. Future studies should be aware of this difference.

Overall morbidity rate was 64 % and mortality rate after pancreatoduodenectomy was 4.6 %. These rates seems slightly higher compared to the contemporary literature [43–46]. This can be explained by the extended inclusion period from 1992 to 2013. The number of operated patients increased during this inclusion period; with the centralization of pancreatic surgery, morbidity rates will also be reduced [47]. The rate of exocrine insufficiency was relatively high with 55 % since other studies report a incidence between 17 and 43 % [18, 48, 49]. This can be explained by the use of different definitions of exocrine insufficiency and the prolonged survival in patients with pNET.

The pancreatic fistula rate of 18 % after pancreatoduodenectomy in this study is slightly higher than the 2–15 % rates reported in literature [5, 8, 50, 51]. This could easily be explained by the reassessment of the complications by an independent researcher according the ISGPS criteria and Clavien–Dindo grading system. Furthermore, the increased rate of pancreatic fistula may be explained by the texture of the pancreatic remnant. Most of our patients undergoing PD and distal pancreatectomy for small pNET had a soft gland with small MPD. The studies with a lower rate of pancreatic fistula have included patients with different types of pancreatic neoplasm or even chronic pancreatitis while a neuroendocrine tumor itself is a risk factor for pancreatic fistula [8, 37]. In addition, enucleation of pNET located 2–3 mm distant from the main pancreatic duct is a risk factor for pancreatic fistula [52, 53]. During tumor enucleation, we attempt to preserve the pancreatic duct. Nevertheless, pancreatic fistula is the major cause for postoperative morbidity in patients with pNETs. In our study, tumor enucleation was not performed in deeply located pNET because these tend to have a close relationship with the main pancreatic duct (MPD). Also, size of the MPD was not predictive for the occurrence of PF, most likely due to the fact that in only 3/60 enucleated patients, the MPD was dilated (Tables 1 and 3). Data regarding the exact relationship between tumor and MPD was not available and an intraoperative ultrasound to measure the distance between MPD, and the tumor was not routinely performed in all patients. However, we do realize that the distance between the tumor and MPD could affect the PF rate. It is a challenge to reduce the rate of pancreatic fistula after pancreatic enucleation. Different techniques with varying success rates are discussed in the literature, such as a teres hepatis ligament flap plasty to cover the enucleation site, placement of an internal pancreatic duct stent or prophylactic use of somatostatin analogs [54–58]. Despite all these efforts, pancreatic fistula remains a problem after pancreatic resections and particularly in patients with pNET.

The present study is one of the largest studies in patients with pNET in which postoperative outcomes were compared between pancreatic enucleation and standard pancreatic resection. However, a limitation of this study is its retrospective design. The choice of the type of resection is made by the surgeon after multidisciplinary consultation and may be influenced by tumor size or other factors. Furthermore, the study period was relatively long encompassing a period of 21 years. Potential underreporting of pancreatic fistula/DGE/postoperative bleeding grade A may have occurred in the early years of the study period. The course of these complications is mild and therefore they are not always explicitly recorded in the discharge letters or patient records. The accuracy of scoring of postoperative complications, especially pancreatic fistula, has increased in recent years with the implementation of the ISGPS criteria. In order to reconcile, all the complications with the current standards, not only the discharge letter but also the entire patient record including laboratory results, medication list, and radiology results was screened for postoperative complications. Furthermore, the rate of pancreatic insufficiency should be carefully interpreted. Because of the retrospective character of the study, it was not always possible to determine whether the patients had developed endocrine insufficiency because of their surgery or due to other causes. Also the number of patients with exocrine insufficiency may have been slightly underestimated since the use of pancreatic enzymes will not always be documented accurately and no objective tests like elastase-1 in the stool was performed.

Since this study was not randomized, the choice for a tumor enucleation or resection was made by the surgeon. Therefore, selection bias may have occurred in the choice of the pancreatic operation. As depicted in Table 1, some patients and tumor characteristics were not comparable between the groups, such as the diameter of the pancreatic duct. An enucleation is often performed in patients with a small pancreatic duct (Table 1) which can increase the incidence of pancreatic fistula, especially in the head of the pancreas. The conversion rate of laparoscopic resection was relatively high because of the learning curve of the surgeons [59]. In the patients with a converted distal pancreatectomy, the tumor was small or challenging to localize. Another limitation of this study is that we did not perform a standard lymph node resection in patients undergoing enucleation. Even in tumors smaller than 2 cm located in the pancreatic head, we found lymph node metastasis in 6 of the 11 patients (55 %). Since no information was available about the presence of lymph node metastasis in patients after tumor enucleation located in the pancreatic head, it was not possible to determine the exact number of metastasis in these small tumors. In this study, R1 resection margin was defined as tumors with microscopic margin involvement <1 mm, not only at the pancreatic resection margin but also at the anterior and posterior margin as well as the median margin close to the portal vein and mesenteric artery. Patients with unclear margins caused by coagulation damage were also scored as R1. Since these strict criteria, the incidence of patients with a positive margin after distal pancreatectomy and pancreatoduodenectomy was relatively high (17 %) compared to studies with other criteria [60].

In conclusion, a comparison was made between enucleation versus pancreatoduodenectomy or distal pancreatectomy to illustrate the differences in outcomes. Postoperative morbidity after enucleation of pancreatic neuroendocrine tumors was comparable to a pancreatoduodenectomy or distal pancreatectomy and therefore it is not to be considered an easy, low-risk operation. In addition, the presence of lymph node metastasis was high for small tumors located in the pancreatic head and 19 % of the NF-pNET patients developed recurrent disease after tumor enucleation. Therefore, a pancreatoduodenectomy might be the preferred operation for most pNET that reside in the head of the gland except for superficially located insulinomas that typically have low malignant potential. In addition, a formal resection of corpus/tail tumors may also be more desirable than enucleation of certain tumors. The data from this study may guide surgeons in selecting appropriate operations for pancreatic neuroendocrine tumors, based upon size, location, and functional status.
